# Onset of Type 2 diabetes in adults aged 50 and older in Europe: an intersectional multilevel analysis of individual heterogeneity and discriminatory accuracy

**DOI:** 10.1186/s13098-024-01533-3

**Published:** 2024-11-29

**Authors:** Julie Lorraine O’Sullivan, Enrique Alonso-Perez, Francesca Färber, Georg Fuellen, Henrik Rudolf, Jan Paul Heisig, Michaela Kreyenfeld, Paul Gellert

**Affiliations:** 1 Institute for Medical Sociology and Rehabilitation Science, Charité – Universitätsmedizin Berlin, Corporate Member of Freie Universität Berlin, Humboldt-Universität zu Berlin, and Berlin Institute of Health, Charitéplatz 1, Berlin, 10117 Germany; 2 Institute for Biostatistics and Informatics in Medicine and Ageing Research, Rostock University Medical Center, Rostock, Germany; 3 WZB Berlin Social Science Center, Berlin, Germany; 4 Freie Universität Berlin, Berlin, Germany; 5 Hertie School Berlin, Berlin, Germany; 6 German Center for Mental Health (DZPG), Berlin-Potsdam, Germany; 7 Einstein Center Population Diversity (ECPD), Berlin, Germany

**Keywords:** Social determinants of health, Health equity, Risk of type 2 diabetes, Intersectionality, Prevention of type 2 diabetes

## Abstract

**Background:**

Disparities in the development of Type 2 Diabetes (T2D) are associated with various social determinants, including sex/gender, migration background, living arrangement, education, and household income. This study applied an intersectional perspective to map social disparities and investigate intersectional effects regarding the onset of T2D among older adults across Europe.

**Methods:**

We used data from the Survey of Health and Retirement in Europe (SHARE) to conduct an Intersectional Multilevel Analysis of Individual Heterogeneity and Discriminatory Accuracy (I-MAIHDA) of T2D onset. Individuals aged 50 years or older without known T2D at Wave 4 (2011, baseline) were included and followed through Waves 5 (2013), 6 (2015), 7 (2016), and 8 (2019–2020). Intersectional models were used to estimate additive main effects of sex/gender, migration background, living arrangement, education level, and household income and intersectional interactions.

**Results:**

A total of 39,108 individuals were included (age at baseline M = 65.18 years (SD = 9.62), 57.4% women). T2D onset was reported for 9.2% of the sample over the 9-year observation period. In the fully adjusted model, all social determinants showed significant additive associations with T2D onset, while the discriminatory accuracy of the social strata was found to be low (Variance Partition Coefficient = 0.3%).

**Conclusions:**

This study provides a comprehensive mapping of intersectional disparities in onset of T2D among older adults in Europe. The results highlight the risk heterogeneity within the population and show social disadvantages faced by certain groups. However, while the T2D risks were higher in some strata than in others, the intersectional effects were small overall and mostly attributable to the additive main effects. The results suggest that public health strategies to prevent T2D should be universal but tailored to meet the specific situation of the different intersectional strata.

**Supplementary Information:**

The online version contains supplementary material available at 10.1186/s13098-024-01533-3.

## Background

According to the World Health Organization, the global increase of Type 2 Diabetes (T2D) in recent decades is a serious public health crisis with a profound impact on societies and health care systems worldwide [1]. T2D is associated with numerous severe health complications, such as an increased risk of cardiovascular disease [2], and is among the six most important drivers leading to an increase in disability-adjusted life years in older adults [3]. In Europe, it is estimated that 8.8% of all the population aged 20–79 years have T2D [4] and the International Diabetes Federation (IDF) projects that T2D prevalence will increase over the next decade [5]. As symptoms can go unrecognized until complications arise, it is further estimated that half of the people with T2D have not yet been diagnosed [4]. Taken together, these rising trends in diagnosed and undiagnosed T2D call for preventative strategies to identify and target those at risk.

T2D usually occurs in middle to old age and is associated with lifestyle-related risk factors such as obesity, a sedentary lifestyle, and smoking [4, 6]. Moreover, sex/gender [7], ethnicity [8] and socioeconomic position [9] have been established as major sociodemographic determinants of T2D. For example, Reus-Pons et al. [10] investigated differences in transition to T2D in migrants compared to non-migrants using data from the Survey of Health, Ageing and Retirement in Europe (SHARE) and found a substantially higher risk of developing T2D in older non-western migrants compared to older non-migrants and in western female migrants compared to non-migrant women.

So far, however, most quantitative studies have not taken an intersectional perspective on social determinants of T2D but rather focused on the additive impact of a single characteristic or specific interactions between a small set of demographic or socioeconomic characteristics [11]. Intersectionality is a theoretical framework based on the view that individual human experiences are collectively shaped by multiple overlapping social identities (e.g., sex/gender, race, class) [12]. These embodied social positions give rise to specific contexts of privilege and disadvantage, and their complex interplay needs to be holistically considered to identify their full impact on health or other outcomes [13]. By considering embodied social identities, the focus shifts from individual characteristics such as sex/gender to contextual conditions. For instance, this allows researchers to acknowledge that the experience of being a woman may differ across social groups, thus uncovering unique inequalities for subgroups at the intersections of sociodemographic categories [14].

Recently, Multilevel Analysis of Individual Heterogeneity and Discriminatory Accuracy (MAIHDA) has been proposed as an innovative approach for incorporating intersectionality in quantitative health research [13, 15]. MAIHDA is a general analytical approach, that can be applied to model inequalities within an intersectional framework [16]. The method was introduced more than two decades ago by Juan Merlo [17] and has since gained increasing recognition as the new gold standard for quantitative intersectional analysis of inequalities in health and other domains [16]. Essentially, the application of Intersectional MAIHDA (I-MAIHDA) allows individual health outcomes (e.g., onset of T2D) to be modeled using a specific form of multilevel regression that treats individuals as nested in social strata defined by the intersections of multiple demographic and socioeconomic characteristics [18]. This approach allows the investigation of heterogeneity within populations while still considering individual social identities [13, 19]. By systematically considering both differences between group averages and individual heterogeneity around those values the “*tyranny of the averages*” (i.e., attribution of the same average value to all members of a certain group) can be avoided [20, 21]. Therefore, I-MAIHDA reduces the risk of reinforcing stereotypes or stigmatizing certain groups. Besides providing a comprehensive mapping of risk heterogeneity across different groups, I-MAIHDA can also be used to disentangle additive from intersectional interactive effects [13, 19]. That is, I-MAIHDA allows us to investigate how individual social determinants contribute to the risk of a certain health risk or disease, while also considering their interactive (multiplicative) contribution.

As for the risk of developing T2D, Wemrell et al. [22] were the first to apply an intersectional approach to investigate demographic and socioeconomic determinants of T2D risk using data from a population-based Swedish health register. Given their large dataset with 4.3 million people, the authors were able to include 120 intersectional strata defined by age, gender, income, education, and immigrant status as fixed effects instead of applying multilevel modeling which has advantages when group sizes are small. Discriminatory accuracy was determined by comparing the area under the receiver operating characteristic curve (AUC), with a focus on the comparison between a model containing the stratum-defining variables only additively and a model that additional included the 120 intersectional strata. Findings revealed substantial socioeconomic heterogeneity in the risk of T2D, underpinning the importance of intersectional strata for understanding the complexity of inequalities in T2D.

## Aims of the present study

In the present study, we aim to expand this research by applying I-MAIHDA to explore intersectional disparities in the onset of T2D in adults aged 50 and older across a diverse set of European countries, with strata defined by the unique combinations of sex/gender, migration background, living arrangement, education level and household income. Our analysis contributes to a better understanding of the interplay of key social categories in shaping the risk of T2D in European societies. It can inform future public health strategies and help identify population groups with high-risk profiles that are in need of targeted prevention strategies.

## Methods

### Study population and database

**Fig. 1:**
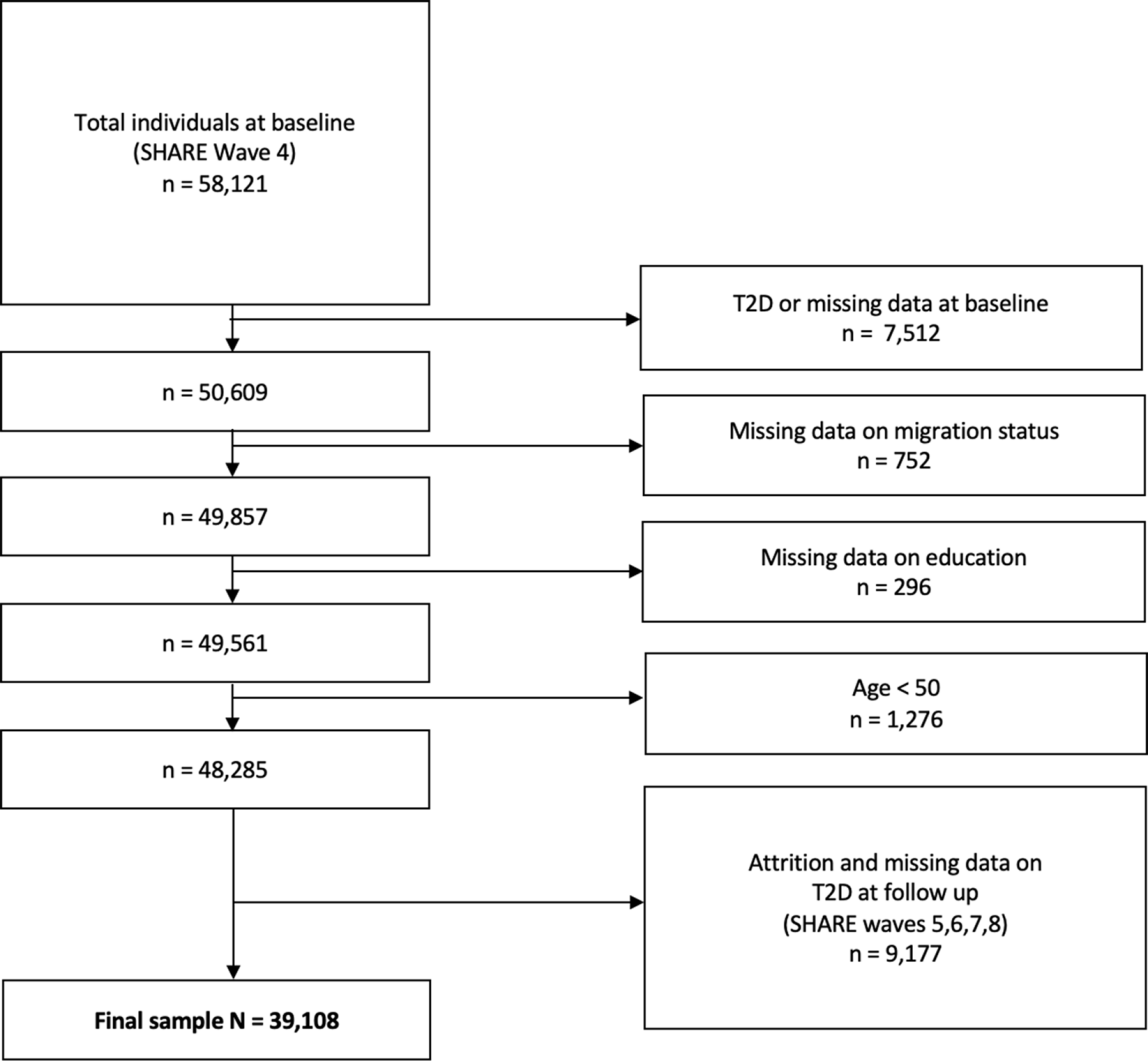
Study population flowchart

We used data from SHARE, which is the largest pan-European social science panel study, providing cross-disciplinary longitudinal data on demographic, socioeconomic, and health variables for people aged 50 years or older and their coresidential partners (16). SHARE data are collected biannually through computer-assisted personal face-to-face interviews (CAPI), and the survey has been extensively described elsewhere [23]. The SHARE questionnaires were revised and modified from Wave 4 onwards [24], meaning that some measures cannot be directly compared with those from Waves (1–3). Hence, we used Wave 4 (2011) as the baseline. We then followed individuals over all subsequent Waves that were available at the time of analysis (i.e., Waves 5, 6, 7, and 8 conducted in 2013, 2015, 2016, and 2019–2020, respectively), resulting in a follow-up period of approximately 9 years (2011–2020). All SHARE respondents aged 50 years and older, without known T2D or other diabetes diagnosis in Wave 4, and without missing data on social strata variables at baseline were included in the analysis (Fig. 1). The initial sample comprised 58,121 individuals^1^, of whom 7,197 (12.4%) already had an existing T2D diagnosis at baseline (see Supplementary Table S1 for characteristics of individuals with existing T2D at baseline). To examine the longitudinal onset of T2D, only respondents without T2D at baseline and with available data at follow-up were included in the analysis. In order to minimize the number of observations lost to follow-up, we included respondents with available data on presence of T2D on any of the follow-up Waves [10]. The final analysis sample consisted of *N* = 39,108 respondents who were at risk of developing T2D. In case of conflicting information on consecutive follow-up Waves (i.e., a present diagnosis recorded for an intermediate Wave but not for the following consecutive Wave), the presence of self-reported diabetes on any Wave was coded as onset of T2D. Conflicting information was found for *n* = 1,020 cases (28% of those with T2D).

The SHARE unique participant identifier was used to link observations from the same participant across survey Waves. Figure 1 provides an overview of study population flow. SHARE was granted ethics approval by the Ethics Council of the Max-Planck-Society.

### Outcome variable

Based on the question “*Has a doctor ever told you that you had any diabetes or high blood sugar*?” in follow-up Waves 5, 6, 7 and 8, the dichotomous outcome variable *onset of T2D* (0 = no onset of T2D; 1 = onset of T2D) was computed for all respondents at follow-up.

### Intersectional strata

We generated 72 (= 2 × 2 × 2 × 3 × 3) intersectional strata based on the combinations of sex/gender (2 levels), migration background (2 levels), living arrangement (2 levels), education level (3 levels) and household income (3 levels): *sex/gender*, *migration background*, *living arrangement*,* education level* and *household income*. These categories were chosen based on known social determinants of T2D [25]. Sex/gender was coded as male or female. Migration background was defined by country of birth: those who were born in their current country of residence were categorized as non-migrants, those who were born in a different country were categorized as migrants. Living arrangement was defined dichotomously as living alone or sharing a household with at least one other person such as a spouse, partner or other family member. Education level was classified according to the International Standard Classification of Education (ISCED 1997) and coded into three categories: high (ISECD 1997 level 5–6), mid-level (ISECD 1997 level 3–4) or low education (ISECD 1997 level 0–2). Finally, tertile values of the SHARE household income variable were used to categorize household income as highest income group, medium income group or lowest income group. We calculated the tertile values based on the data available for the total sample in SHARE Wave 4. The household income was either directly reported by respondents, or in cases of missing data, one of the available multiple imputations in SHARE was used, according to the methodology described elsewhere [26]. We adjusted the income that was provided in national currencies in the data by purchasing power parity (PPP) exchange rates in order to allow cross-sectional country comparisons of financial variables, with Germany as a reference country. As not all respondents in Wave 4 were selected in our final sample (i.e., due to missing data), the tertile values do not reflect equal thirds of the sample but rather correspond to the actual income position within the interview country. Overall, *n* = 13,539 (34,6%) of the final sample were assigned to the highest income group, *n* = 13,015 (33,3%) were assigned to the medium income group and *n* = 12,554 (32,1%) were assigned to the lowest income group.

### Statistical analysis

We performed an I-MAIHDA for the onset of T2D with individual respondents (level 1) nested in social strata (level 2). Following the procedure developed by Evans and Merlo [13, 15, 16, 27], the onset of T2D was analysed through three successive multilevel logistic regression models described below. We also calculated the AUC as a well-established measure of discriminatory accuracy in clinical epidemiology [28]. All analyses were run in Stata/BE^®^18.0 (Statacorp, College Station, TX, USA). P-values < 0.05 (two-tailed) were considered statistically significant.

### Model 1: unadjusted intersectional model

In a first step, the simple intersectional model included only an intercept and random effect for the social strata (null model). This model provides information on the overall inequality in the sample by producing stratum-specific predictions of T2D onset and summarizing the degree of heterogeneity within and between strata. The outcome predicted onset of T2D and 95% confidence intervals (95%CI) were estimated for each of the 72 social strata based on model 1. No covariates were included in the null model as it was used to conduct a simple analysis of the individual variance components (i.e., between and within-strata variance) and to compute the Variance Partition Coefficient (VPC), often also referred to as the Intraclass Correlation Coeffcient (ICC). The VPC provides an estimate of the variance in T2D onset that lies *between* strata. A higher VPC indicates a higher degree of clustering of T2D onset within strata, i.e., greater similarity in T2D onset within the strata and greater differences across the strata. The proportion of T2D variation that lies *within* the strata is indicated by 1-VPC.

A challenge in estimating the VPC in multilevel logistic regression is that, in contrast to multilevel linear regression with continuous outcomes, the level-1 (i.e., respondent-level residuals) cannot be estimated directly. We adopt the widely used approach based on the latent response formulation of the model and estimate the VPC as follows [29]:

$$\:VPC=\:\frac{{\begin{array}{c}{\sigma\:}_{u}\end{array}}^{2}}{\begin{array}{c}{\sigma\:}_{u}^{2}+3.29\end{array}}\:\times\:\:100$$,

where multiplication by 100 allows for interpretation in percentage terms. In this equation, $$\:{\begin{array}{c}{\sigma\:}_{u}\end{array}}^{2\:}$$denotes the between-stratum variance in the onset of T2D, while 3.29 denotes the within-strata between-individual variance constrained equal to the variance of the standard logistic distribution [29]. This model was also intended to determine the predicted T2D onset for each of the intersectional strata. Since the probability scale favours interpretation, the predicted logit (log-odds) of developing T2D were transformed into the probability of developing T2D for every intersectional stratum [30].

As recently pointed out by Axelsson Fisk and colleagues [30], there is no unified classification system for interpretation of VPC values in social epidemiology. However, based on the widely accepted grading of Intraclass Coefficients (ICC), the authors propose the following classification of discriminatory accuracy: non-existent (0–1), poor (> 1 to ≤ 5), fair (> 5 to ≤ 10), good (> 10 to ≤ 20), very good (> 20 to ≤ 30), excellent (> 30).

### Model 2: main effects model

In the main effects model, all social strata variables are included additively (sex/gender, migration background, living arrangement, education level and household income) as fixed effects. Odds ratios (ORs) and 95%CI were also estimated for the strata variables (i.e. sex/gender, migration background, living arrangement, education level and household income), with ORs above 1 indicating an increased chance of developing T2D at follow-up whereas ORs below 0 indicate a reduced chance. The Proportional Change in Variance (PCV) was calculated to quantify the proportion of the stratum-level variance from the unadjusted intersectional model that is explained by the additive main effects. The PCV was calculated as:

$$\:PCV=\:\frac{\begin{array}{c}\begin{array}{c}\begin{array}{c}{\sigma\:}_{u\left(1\right)}^{2}-\:{\sigma\:}_{u\left(2\right)}^{2}\end{array}\end{array}\end{array}}{{\sigma\:}_{u\left(1\right)}^{2}}\:\times\:\:100$$

In the PCV equation, $\:{\sigma\:}_{u\left(1\right)}^{2}$ and $\:{\sigma\:}_{u\left(2\right)}^{2}$ denote the between stratum variance derived from models 1 and 2. The PCV was multiplied by 100 to obtain percentages. A high PCV indicates that most of the stratum-level variance is explained by the additive main effects, while a low PCV indicates that it is explained by multiplicative between-strata interactions, i.e., by intersectional effects [31]. Furthermore, we obtained estimates of stratum random effects to measure stratum-specific risk and identify strata with higher and lower T2D risk than expected based on the additive main effects only. This is done by decomposing the absolute risk of T2D into two parts: (1) risk of T2D explained by the main effects and (2) risk of T2D explained by higher order interaction effects between the included variables. The random effects (interactions) of each stratum allow us to assess the presence and magnitude of such stratum-specific hazardous or protective interaction effects [19]. For the purpose of a sensitivity analysis, we calculated an additional intersectional model including all the social strata variables and adjusted for age and country. The PCV and estimates of T2D risk at the stratum-level based on model 3 are provided in the supplementary material (supplementary Tables S2 and S3).

## Results

Overall, onset of T2D was observed in *n* = 3,609 respondents (9.23%) at follow-up (SHARE Waves 5,6,7,8). At baseline, participants average age was M = 65.18 years (SD = 9.62), and *n* = 22,449 (57.40%) were women. A total of *n* = 3,597 (9.20%) reported migration background. The majority of participants were living in a shared household *n* = 30,727 (78.57%). Most of the sample had low *n* = 15,592 (39.87%) or mid-level *n* = 15,227 (38.94%) education, while high education was reported for *n* = 8,289 (21.20%) participants. Regarding household income, *n* = 13,359 (34.62%) participants were assigned to the group with the highest income. Table 1 provides an overview of baseline characteristics by T2D onset for follow-up.

**Table 1:** Characteristics of the study sample according to diabetes status at the end of follow up, *N* = 39,108)

	Diabetes status at follow-up			
	Diabetes		No Diabetes	
Characteristics at baseline				
Total	3,609	9.23	35,499	90.77
Sex/Gender				
Men n, %	1,679	46.52	14,980	42.20
Women n, %	1,930	53.48	20,519	57.80
Migration background				
No migration background n, %	3,219	89.19	32,292	90.97
Migration background n, %	390	10.81	3,207	9.03
Living arrangement				
Cohabiting n, %	2,797	77.50	27,930	78.68
Living alone n, %	812	22.50	7,569	21.32
Education (ISCED)				
High education n, %	516	14.30	7,773	21.90
Mid-level education n, %	1,308	36.24	13,919	39.21
Low education n, %	1,785	49.46	13,807	38.89
Household net income				
High income n, %	1,005	27.85	12,534	35.31
Medium income n, %	1,089	30.17	11,926	33.60
Low income n, %	1,515	41.98	11,039	31.10
Covariates				
Age, mean (SD)	66.42 (9.08)		65.06 (9.67)	

Note: ISCED = International Standard Classification of Education; SD = Standard Deviation

The VPC of 4.3% (95% CI: 2.7–6.6) in the unadjusted intersectional model can be classified as poor [30], meaning that only a small portion of the individual variability in T2D onset can be explained at the intersectional strata level (Table 2). This is confirmed by an AUC of 0.61 (95% CI: 0.60–0.62), commonly considered as a very low level of discriminatory accuracy [32]. This VPC value represents the total possible effect that can be attributed to the social strata variables. In the intersectional interaction model including each of the strata variables as fixed main effects (model 2), the VPC dropped to 0.3% (95% CI: 0.1–1.1) and the AUC dropped to 0.60 (95% CI: 0.59–0.61), indicating that most of the differences in individual T2D onset can be explained by the additive main effects of sex/gender, migration background, living arrangement, education level and household income. Accordingly, the PCV from model 1 to model 2 was 92.2%. Women had a lower chance of developing T2D compared to men (OR = 0.79, 95% CI: 0.71–0.87). Having a migration background compared to non-migrants (OR = 1.23, 95% CI: 1.09–1.40), living alone compared to not living alone (OR = 1.13, 95% CI: 1.01–1.26) having low education (OR = 1.78, 95% CI: 1.56–2.04) or mid-level education (OR = 1.34, 95% CI: 1.17–1.54) compared to high education level, and having low compared to high household income (OR = 1.57, 95% CI: 1.39–1.77) were associated with higher odds of T2D onset during follow up.

**Table 2:**
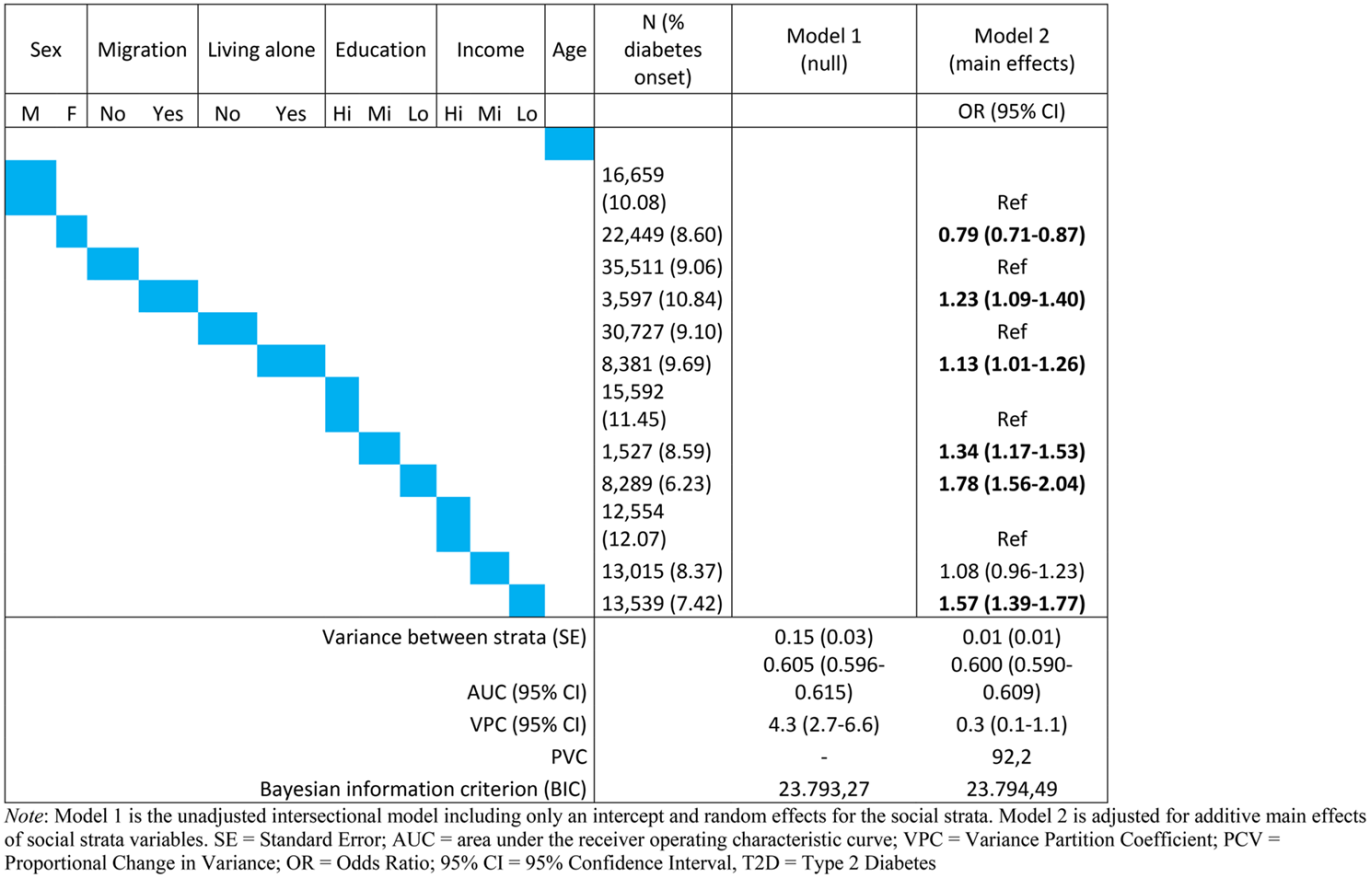
Results of the multilevel logistic regression models on onset of type-2-diabetes over the 9-year observation period

To put these values in perspective, we also estimated a model with countries as the second level and calculated the VPC and AUC. The results show VPC of 6.2 (95% CI: 3.1–12.0) and an AUC of 0.62 (95% CI: 0.61–0.63), indicating that the discriminatory accuracy of the included eleven countries is low.

**Fig. 2:**
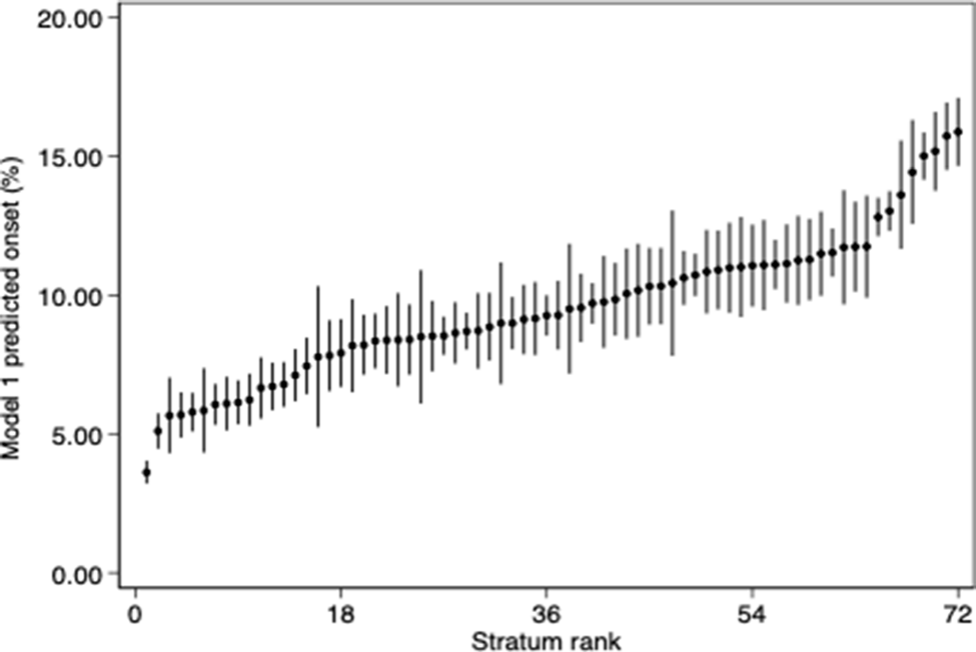
Predicted onset of type-2-diabetes over the 9-year observation period by intersectional strata

Out of all 72 intersectional strata, the stratum with the lowest onset of T2D predicted by model 1 was female, non-migrant, cohabiting with high education and high income (3.61%, 95% CI: 3.20–4.03), see Table 3. In contrast, the stratum with the highest predicted onset of T2D was female, migrant, cohabiting with low education and low income (15.88%, 95% CI: 14.65–17.11). Figure 2 shows the predicted onset of T2D over the 9-year observation period for each stratum based on predictions from the unadjusted model.

**Table 3:**
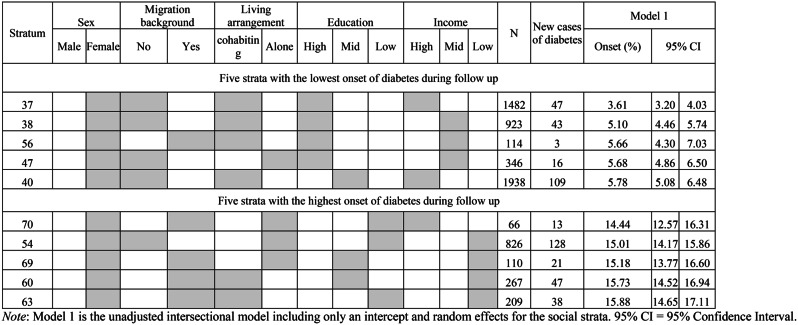
Observed onset of type-2-diabetes and predictions based on model 1 over the 9-year observation period by intersectional strata

**Fig. 3:**
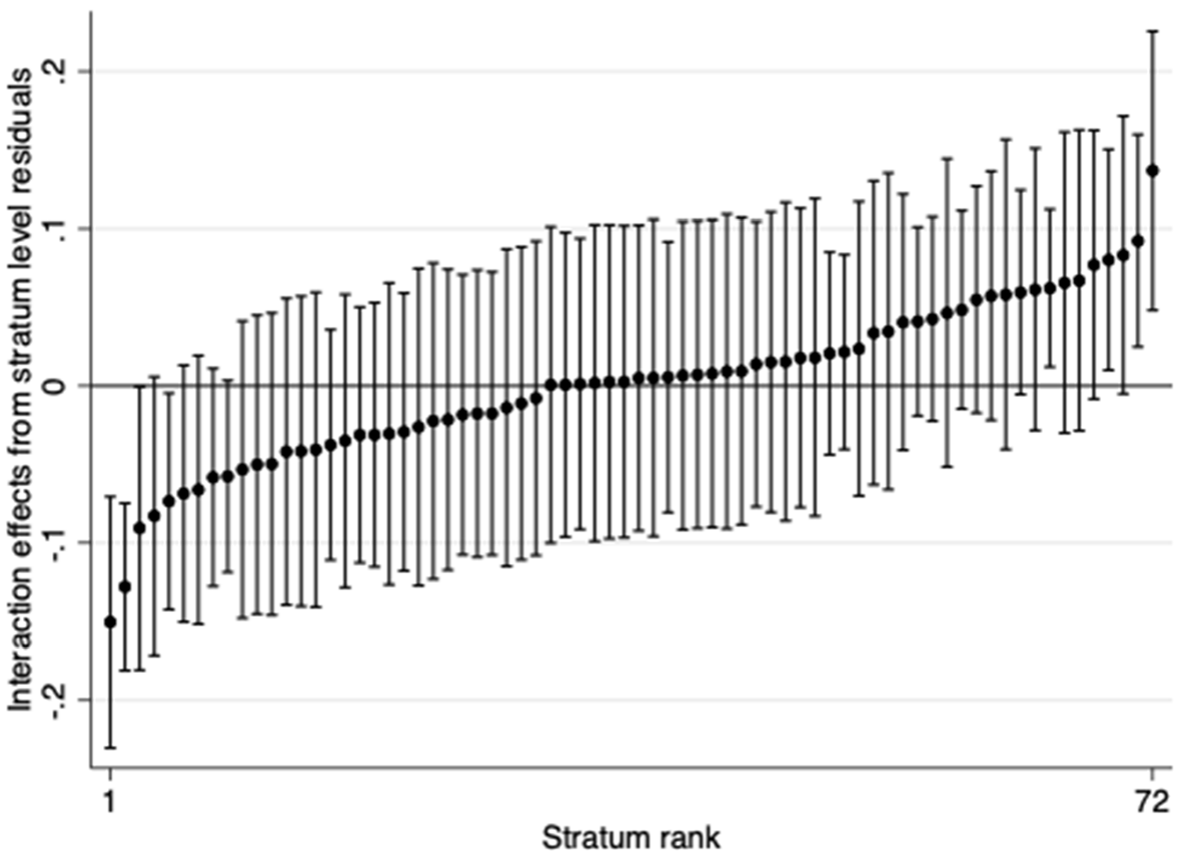
Differences in 9-year diabetes onset due to interaction effects (i.e., stratum level residuals with 95% confidence intervals from model 2)

Table 4 shows the interaction effects for each of the 72 strata (for a discussion of multiplicative interactions in the context of MAIHDA, please see [19, 33]). Only seven strata have statistically significant multiplicative interactional effects, which is in line with the low VPC observed in the fully adjusted intersectional model. Protective effects were observed for four strata: women with migration background who do not live alone with high education and low household income levels (interaction effect = -0.15, 95% CI: -0.23 - -0.07), women without migration background who do not live alone with mid education and low household income levels (interaction effect = -0.13, 95% CI: -0.18 - -0.07), women without migration background who live alone with mid education and mid household income levels (interaction effect = -0.09, 95% CI: -0.18 - -0.01) and women with migration background who do not live alone with low education and high income (interaction effect = -0.07, 95% CI: -0.14 - -0.01). On the other hand, three strata had significant hazardous effects: men with migration background who live alone with high education and mid household income levels (interaction effect = 0.08, 95% CI: 0.01–1.15), men without migration background who live alone with mid education and high household income levels (interaction effect = 0.09, 95% CI: 0.02–0.16), and women with migration background who do not live alone and have high education and high household income levels (interaction effect = 0.14, 95% CI: 0.05–0.23). Figure 3 shows the stratum level residuals for each stratum based on predictions from model 2.

**Table 4:**
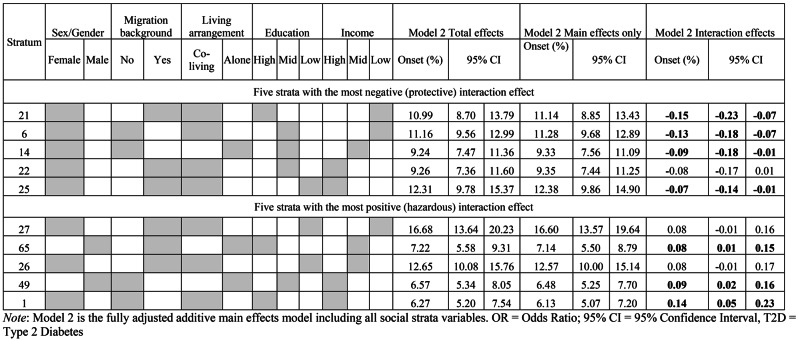
Predicted type-2-diabetes onset over the 9-year observation period based on the total effect (intersectional effects and main effects) and main effects only (model 2)

## Discussion

In the present study, we applied I-MAIHDA to analyze inequalities in the onset of T2D in adults aged 50 years and older using data from SHARE. Respondents were assigned to 72 intersectional social strata based on the combination of social categories sex/gender, migration, living arrangement, education and household income. While the results showed noteworthy additive effects of the social determinants on onset of T2D, the multiplicative interactional effects on onset of T2D were found to be limited. Although social determinants play an important role regarding the risk of T2D in adults aged 50 and older, we found limited evidence for an amplified or attenuated risk in certain groups based on their social identities. This finding is in line with previous I-MAIHDA studies investigating multiple health outcomes [34]. Taken together, our findings underscore the importance of social determinants regarding the risk of T2D, although we did not observe prominent intersectional effects. That is, risk of T2D was observed across the whole population and not only in specific intersectional strata.

The present study revealed a notable cumulative incidence of T2D among older adults in Europe, with 9.23% of the respondents developing T2D during the 9-year follow-up period. These findings align with the global increase of T2D and highlight the urgent need for preventive strategies to identify and target those at risk [35]. In this regard, the presented results confirm the importance of social determinants of T2D, such as sex/gender, migration background, and living arrangement, education level and household income, which have been previously established in the literature [7–9]. Regarding sex/gender differences in T2D, a recent review reported similar findings, revealing a higher prevalence of T2D diagnosis observed in men [36]. However, due to changes in sex hormones across the lifetime, women experience greater variations in their T2D risk, and it has been observed that women with T2D face a heightened susceptibility to cardiovascular complications associated with T2D. Moreover, gendered factors such as health behavior, lifestyle and attitudes towards prevention and treatment play a central role in the context of prevention and management of T2D [37]. Given the distinct risks and health consequences associated with T2D in men and women, our findings also support the need for gender specific T2D prevention strategies. Physicians and healthcare professionals should be aware of gendered risks regarding T2D and advise their patients accordingly. Interestingly, our results reveal noteworthy patterns regarding sex/gender and T2D onset during the follow-up period. While female gender was associated with a lower risk of developing T2D, we observed that both the five strata with the highest and lowest T2D onset at follow up were women. Interestingly, those with the lowest risk of developing T2D were predominantly women without a migration background and with higher levels of education. Conversely, those with the highest T2D onset were women mostly with migration background and mid- or low-level education. These results underline the importance of considering the interplay of sex/gender with further social identities to better understand health vulnerabilities in multifaceted populations.

In line with our results, disparities in T2D have previously been reported among individuals with a migration background in Europe [38]. Studies have reported higher T2D incidence and prevalence among first generation migrants compared to non-migrants. However, the extent of T2D risk appears to vary depending on the region of origin, with populations from South Asia, the Middle East, and North Africa being particularly vulnerable [38, 39]. These findings underscore the importance of considering ethnicity and migration background in a nuanced manner, to gain a deeper understanding of implications regarding the risk and onset of T2D. Risk profiles may vary based on factors such as country of origin, country of residence, and generation of migration, thus calling for further research to identify communities at risk and develop targeted prevention strategies.

We also found an increased T2D risk in individuals living alone compared to those who live with others. In line with this finding, a previous study using data from a nationwide cohort study in Korea also identified living alone as a risk factor for T2D [40]. Living alone may increase psychological stress and feelings of loneliness, which have been established as risk factors for T2D [41]. Furthermore, living alone may lead to social isolation and result in reduced access to social and emotional support networks, which are crucial for maintaining overall health and well-being [42]. As European societies are becoming more diverse in terms of living arrangements with the number of people living in one-person households increasing rapidly [43] more research is needed on strategies to reduce loneliness and social isolation in those living alone. Importantly, Nam et al. [40] found that the associations between living alone and T2D differed by age and sex/gender, with stronger associations in men and younger individuals. Furthermore, it has been pointed out that older men living in one-person households seem to be a particularly high risk group when it comes to health and health-care utilization [44]. These findings further underscore the relevance of taking an intersectional perspective when considering the role of social determinants of T2D and health in general.

Our results revealed associations of both educational level and household income with T2D onset. Previous research has also highlighted disparities in T2D associated with socioeconomic status (SES) [45, 46]. Education and household income are two crucial components of SES, thus our results confirm previous findings linking SES to the development of T2D. Individuals with lower educational attainment and economic resources often face barriers in accessing quality healthcare, health information, and resources necessary for T2D prevention and management [47]. As pointed out by Blanquet et al. [46], cardio-metabolic disease prevention campaigns targeting the general population often do not meet the needs of groups with high social or economic vulnerability. In light of these socioeconomic disparities, our results emphasize the need for promoting health literacy and improving healthcare access in disadvantaged populations. By using intersectionality-informed approaches such as MAIHDA to map health outcomes across different population groups, researchers can accurately identify those subgroups who may be especially in need of targeted prevention strategies. These strategies can contribute to mitigating the impact of socioeconomic disparities and reducing the overall burden of T2D.

### Strengths and limitations

As most previous quantitative studies have focused on examining the sole impact and interactions of individual social determinants of T2D, our study adds to this research by taking an intersectional perspective. Intersectionality acknowledges that individual human experiences are collectively shaped by multiple overlapping social identities, such as gender, race, and class. It emphasizes the need to consider systemic structural inequalities related to contextual conditions [14]. By considering both additive and multiplicative (i.e., intersectional) effects, our analysis incorporates an approach that is aligned with the notion of recognizing and addressing the complex interplay of multiple social determinants in understanding health disparities. This comprehensive perspective allows for a more nuanced understanding of how various social identities interact to shape the incidence of T2D among older adults in Europe. Even though the present study found absence of evidence for interactional multiplicative effects regarding the onset of T2D, future studies should apply this approach to gain a deeper understanding of the social contexts that shape health and well-being.

Taken together, our results emphasize that risk of T2D affects the whole population and not only specific strata, as reflected by the low VPC. Having said that, our results also show that certain strata have a much higher risk (i.e., higher positive predictive value) than others. For instance, the stratum comprising “women, migrant, cohabiting with low education and low income” has a four times higher risk than the stratum of “women, non-migrant, cohabiting with high education and high income”. Therefore, a possible universal prevention approach needs be tailored to the specific characteristics of each stratum. For instance, promoting integration, health literacy and economical resources may reduce the risk of T2D in women with migration background. However, the question remains if the T2D risk in the stratum “women-non-migrant-cohabiting-high education-high income” with the lowest risk (i.e., 3.6%) represents the floor value of T2D risk that cannot be modified by traditional interventions. Similar to an approach applied by Merlo et al. in a study of intersectional inequalities in obesity [32], this value of 3.6% could serve as a predetermined benchmark level for informed prevention strategies. Using intersectionality-informed MAIHDA researchers obtain an improved mapping of risk across different population groups and also can understand if interventions should be universal, universal with tailored components, or targeted. These strategies can contribute to mitigating the impact of socioeconomic disparities and reducing the overall burden of T2D.

Several limitations of the study must be pointed out. Firstly, the T2D diagnosis in this study was based solely on self-reporting and was not validated by laboratory parameters or medical records. Previous research has explored the presence of protective hormetic effects at the molecular level, which seem to mitigate the risk of T2D in some individuals with pronounced lifestyle risk factors [48]. Hormetic effects may have contributed to the finding of diminished multiplicative interactional effects in our study. Future studies should aim to incorporate additional sources of medical information, such as biomarkers like HbA1c or medical records, to validate the diagnosis of T2D and explore possible molecular defense mechanisms. This approach would not only enhance the accuracy of the findings but also enable the identification of individuals who may have undiagnosed T2D. On a related note, due to the reliance on self-reported diagnosis of any kind of diabetes, we cannot rule out that individuals with other diabetes types than T2D may have been included. However, given that T2D is predominantly observed in later stages of life, and in line with previous studies using diabetes self-reports from SHARE, we assume that newly observed cases of diabetes in SHARE were indeed cases of T2D [49]. We also did not consider the possibility of individuals who may have developed T2D but were later cured due to lifestyle modifications or other treatments. Future studies should take a more nuanced look at trajectories of T2D and how these might differ across social positions. The assignment of respondents to the intersectional strata was based on their positions in SHARE Wave 4 (baseline) to investigate the contextual effects of belonging to a certain intersectional stratum over time. Some of the intersectional strata variables can change over time, for instance living arrangement or income. Future studies should take these transitions into account to investigate the effects of social mobility in the context of T2D risk. Furthermore, there was some attrition in sample size over time. It is important to acknowledge that the presence of selection bias cannot be completely ruled out, which may impact the generalizability of the findings. Lastly, it is worth noting that the variable for household income available in SHARE was missing for a number of respondents. To address this issue and mitigate potential bias, we followed SHARE’s recommendation and included SHARE household income with multiple imputation whenever the variable was not available in our analysis. This approach allows us to obtain more robust parameter estimates and minimize the impact of missing data on the findings [50].

## Conclusion

The present study revealed a notable cumulative incidence of T2D among older adults in Europe, emphasizing the urgency of implementing preventive measures to identify and target at-risk individuals. The importance of social determinants, such as sex/gender, migration background, living arrangement, education, and household income, was confirmed, aligning with previous literature. Reducing inequalities in T2D onset requires the implementation of tailored intervention strategies that specifically address the unique needs of at-risk populations. By applying an intersectional perspective, healthcare professionals and public health initiatives can consider the complex interplay of social determinants and reduce health disparities in chronic diseases such as T2D. Intersectionality-informed intervention and prevention strategies are needed to effectively improve access to quality healthcare, health education, resources, and support for disadvantaged groups.

## Electronic supplementary material

Below is the link to the electronic supplementary material.

Supplementary Material 1

## Data Availability

SHARE data is publicly available after free registration (https://share-eric.eu/data/). Materials are available from the corresponding author on reasonable request.

## Footnotes

1 Respondents were from all 11 participating countries in SHARE Wave 4: Austria, Germany, Sweden, Netherlands, Spain, Italy, France, Denmark, Switzerland, Belgium, Czech Republic, Poland, Hungary, Portugal, Slovenia, Estonia.

## Abbreviations

AUC: Area under the receiver operating characteristic curve
CI: Confidence interval
IDF: International Diabetes Federation
I-MAIHDA: Intersectional Multilevel analysis of individual heterogeneity and discriminatory accuracy
ISCED: International Standard Classification of Education
MAIHDA: Multilevel analysis of individual heterogeneity and discriminatory accuracy
OR: Odds ratio
PCV: Proportional Change in Variance
SD: Standard deviation
SE: Standard error
SES: Socioeconomic status
SHARE: Study of Health and Retirement in Europe
T2D: Type 2 Diabetes
VPC: Variance Partition Coefficient
