# Supplementary material for: Onset of Type 2 diabetes in adults aged 50 and older in Europe: an intersectional multilevel analysis of individual heterogeneity and discriminatory accuracy
Source: Diabetol Metab Syndr. 2024 Nov 29;16:291. doi: 10.1186/s13098-024-01533-3 (PMC11605936; doi:10.1186/s13098-024-01533-3)
Supplement: Supplementary file 1 — Supplementary Material 1 [file 13098_2024_1533_MOESM1_ESM.docx]

Supplementary Tables

Supplementary table S1: Characteristics of persons excluded at baseline due to an existing type-2-diabetes diagnosis at baseline, n = 7,197

|  |  |  |
| --- | --- | --- |
|  |  | |
| Characteristics at baseline |  |  |
| Sex |  |  |
| Male n, % | 3,380 | 47.00 |
| Female n, % | 3,817 | 53.00 |
| Migration background |  |  |
| No migration background n, % | 6,350 | 88.20 |
| Migration background n, % | 742 | 10.30 |
| Missing n, % | 105 | 1.5 |
| Living arrangement |  |  |
| Cohabiting n, % | 5,476 | 76.10 |
| Living alone n, % | 1,721 | 23.90 |
| Education (ISCED) |  |  |
| High education n, % | 918 | 12.80 |
| Mid-level education n, % | 2,392 | 33,20 |
| Low education n, % | 3,724 | 51.70 |
| Missing n, % | 163 | 2,3 |
| Household net income |  |  |
| High income n, % | 1,904 | 26.50 |
| Medium income n, % | 2,336 | 32.05 |
| Low income n, % | 2,957 | 41.10 |
| Covariates |  |  |
| Age, mean (SD) | 68.41 (9.56) |  |

*Note*: ISCED = International Standard Classification of Education; SD = Standard Deviation

Supplementary table S2: Results of the multilevel logistic regression models on onset of type-2-diabetes over the 9-year observation period

| Sex | | Migration | | | Living alone | | | | Education | | | | Income | | | Age | N (% diabetes onset) | Model 1 (null) | Model 2 (main effects) | Model 3 (main effets + controls) |
| --- | --- | --- | --- | --- | --- | --- | --- | --- | --- | --- | --- | --- | --- | --- | --- | --- | --- | --- | --- | --- |
| M | F | No | Yes | | No | | Yes | | Hi | | Mi | Lo | Hi | Mi | Lo |  |  |  | OR (95% CI) | OR (95% CI) |
|  |  |  |  |  | |  | |  | | |  |  |  |  |  |  |  |  |  | **1.01 (1.00-1.01)** |
|  |  |  |  |  | |  | |  | | |  |  |  |  |  |  | 16,659 (10.08) |  | Ref | Ref |
|  |  |  |  |  | |  | |  | | |  |  |  |  |  |  | 22,449 (8.60) |  | **0.79 (0.71-0.87)** | **0.79 (0.71-0.87)** |
|  |  |  |  |  | |  | |  | | |  |  |  |  |  |  | 35,511 (9.06) |  | Ref | Ref |
|  |  |  |  |  | |  | |  | | |  |  |  |  |  |  | 3,597 (10.84) |  | **1.23 (1.09-1.40)** | **1.23 (1.08-1.40)** |
|  |  |  |  |  | |  | |  | | |  |  |  |  |  |  | 30,727 (9.10) |  | Ref | Ref |
|  |  |  |  |  | |  | |  | | |  |  |  |  |  |  | 8,381 (9.69) |  | **1.13 (1.01-1.26)** | **1.13 (1.01-1.26)** |
|  |  |  |  |  | |  | |  | | |  |  |  |  |  |  | 15,592 (11.45) |  | Ref | Ref |
|  |  |  |  |  | |  | |  | | |  |  |  |  |  |  | 1,527 (8.59) |  | **1.34 (1.17-1.53)** | **1.34 (1.17-1.54)** |
|  |  |  |  |  | |  | |  | | |  |  |  |  |  |  | 8,289 (6.23) |  | **1.78 (1.56-2.04)** | **1.78 (1.56-2.04)** |
|  |  |  |  |  | |  | |  | | |  |  |  |  |  |  | 12,554 (12.07) |  | Ref | Ref |
|  |  |  |  |  | |  | |  | | |  |  |  |  |  |  | 13,015 (8.37) |  | 1.08 (0.96-1.23) | 1.08 (0.96-1.22) |
|  |  |  |  | |  | | |  | |  |  |  |  |  |  |  | 13,539 (7.42) |  | **1.57 (1.39-1.77)** | **1.57 (1.39-1.78)** |
| Variance between strata (SE) | | | | | | | | | | | | | | | | |  | 0.15 (0.03) | 0.01 (0.01) | 0.01 (0.07) |
| AUC (95% CI) | | | | | | | | | | | | | | | | |  | 0.605 (0.596-0.615) | 0.600 (0.590-0.609) | 0.600 (0.589-0.607) |
| VPC (95% CI) | | | | | | | | | | | | | | | | |  | 4.3 (2.7-6.6) | 0.3 (0.1-1.1) | 0.3 (0.1-1.1) |
| PVC | | | | | | | | | | | | | | | | |  | - | 92,2 | 92,3 |
| Bayesian information criterion (BIC) | | | | | | | | | | | | | | | | |  | 23.793,27 | 23.794,49 | 23.789,75 |

*Note*: Model 1 is the unadjusted intersectional model including only an intercept and random effects for the social strata. Model 3 is adjusted for additive main effects of social strata variables and covariates age and country. Model 2 is the additive main effects model including all social strata variables. SE = Standard Error; AUC = area under the receiver operating characteristic curve; VPC = Variance Partition Coefficient; PCV = Proportional Change in Variance; OR = Odds Ratio; 95% CI = 95% Confidence Interval, T2D = Type 2 Diabetes

Supplementary table S3: Predicted type-2-diabetes onset over the 9-year observation period based on the total effect (intersectional effects and main effects) and main effects only (model 3)

| Stratum | Sex/gender | | Migration background | | Living arrangement | | Education | | | Income | | | Model 3 Total effects | | | Model 3 Main effects only | | Model 3 Interaction effects | | | |
| --- | --- | --- | --- | --- | --- | --- | --- | --- | --- | --- | --- | --- | --- | --- | --- | --- | --- | --- | --- | --- | --- |
|  | Female | Male | No | Yes | Co-living | Alone | High | Mid | Low | High | Mid | Low | Onset (%) | 95% CI | | Onset (%) | 95% CI | Onset (%) | | 95% CI | |
| Five strata with the most negative (protective) interaction effect | | | | | | | | | | | | | | | | | | | | | |
| 37 |  |  |  |  |  |  |  |  |  |  |  |  | 3.23 | 2.76 | 3.69 | 4.67 | 4.20 | 5.15 | **-1.45** | **-2.27** | **-0.62** |
| 9 |  |  |  |  |  |  |  |  |  |  |  |  | 13.81 | 11.92 | 15.70 | 15.10 | 13.37 | 16.84 | **-1.29** | **-2.09** | **-0.50** |
| 27 |  |  |  |  |  |  |  |  |  |  |  |  | 17.18 | 15.02 | 19.33 | 18.10 | 16.13 | 20.07 | -0.92 | -2.00 | 0.16 |
| 24 |  |  |  |  |  |  |  |  |  |  |  |  | 13.29 | 11.53 | 15.04 | 14.12 | 12.50 | 15.74 | -0.83 | -1.76 | 0.09 |
| 41 |  |  |  |  |  |  |  |  |  |  |  |  | 5.98 | 5.22 | 6.73 | 6.70 | 5.97 | 7.44 | **-0.73** | **-1.40** | **-0.05** |
| Five strata with the most positive (hazardous) interaction effect | | | | | | | | | | | | | | | | | | | | | |
| 3 |  |  |  |  |  |  |  |  |  |  |  |  | 9.93 | 8.74 | 11.11 | 9.23 | 8.13 | 10.34 | 0.69 | -0.18 | 1.57 |
| 54 |  |  |  |  |  |  |  |  |  |  |  |  | 14.47 | 12.62 | 16.32 | 13.72 | 12.03 | 15.42 | **0.75** | **0.05** | **1.44** |
| 13 |  |  |  |  |  |  |  |  |  |  |  |  | 9.48 | 8.21 | 10.75 | 8.64 | 7.45 | 9.82 | 0.84 | -0.04 | 1.73 |
| 7 |  |  |  |  |  |  |  |  |  |  |  |  | 11.08 | 9.63 | 12.52 | 10.20 | 8.86 | 11.54 | **0.88** | **0.19** | **1.56** |
| 60 |  |  |  |  |  |  |  |  |  |  |  |  | 12.74 | 11.41 | 14.07 | 11.38 | 10.15 | 12.62 | **1.36** | **0.39** | **2.33** |

*Note*: Model 3 is the fully adjusted additive main effects model including all social strata variables and covariates age and country. OR = Odds Ratio; 95% CI = 95% Confidence Interval, T2D = Type 2 Diabetes
